# Examining Predictors and Outcomes of Decent Work Perception with Chinese Nursing College Students

**DOI:** 10.3390/ijerph17010254

**Published:** 2019-12-30

**Authors:** Yin Ma, Junjer You, Yuanxiong Tang

**Affiliations:** 1 Research Center for Social and Economic Development Assessment, Lanzhou University, Lanzhou 730000, China; 2 Department of Artificial Intelligence, CTBC Business School, Tainan 700, Taiwan; youjunjer@ctbc.edu.tw; 3 School of Philosophy and Sociology, Lanzhou University, Lanzhou 730000, China

**Keywords:** psychology of working theory, future decent work perceptions, career exploration, nursing students

## Abstract

Drawing on the psychology of working theory (PWT), the present study was performed to evaluate the predictor session of the PWT and career exploration behavior with a sample of Chinese nursing college students from diverse backgrounds. The research employed a cross-sectional research design and 854 nursing students were recruited from one health vocational college situated in Northwest China. Structural equation modeling was utilized to conduct the analysis; confirmatory factor analysis and structural model testing were performed accordingly. Moreover, mediation analysis that used bias-corrected percentile bootstrapping method and moderation analysis were carried out in accordance. Overall, most of the proposed direct paths were significant, but the mediation results were mixed. Proactive personality simply moderated the impact of economic constraints on career adaptability. The results suggest that PWT is applicable to nursing college students and the model is generally supported in the Chinese context. It is the first empirical work that used this model among nursing college students and future decent work perceptions contributed another important antecedent of career exploration behavior. The practical implications based on these findings are provided as well.

## 1. Introduction

Career exploration is considered as a vital outcome in the majority of career development literature, and those who successfully display career-related self and environmental exploration may contribute to career decision self-efficacy [1], career-related knowledge and capabilities [2,3] and career identity development [4,5]. For example, by performing the quasi-experimental research design, researchers found that career exploration could effectively promote one’s career decidedness [1]. Similarly, studies found that career exploration is positively associated with career adaptability, which in turn affects an individual’s self-regulation abilities/resources in career development [3]. Moreover, with the passage of time, individuals who engaged in career exploration can define themselves better and devote themselves to self-identification in the career environment [5]. Hence, it is of great significance to understand the antecedents of career exploration, and thus design the appropriate approaches to enhance students’ career exploration behavior.

In counseling psychology, a more inclusive theory has been called for by scholars to better evaluate the career-related aspects of disadvantaged groups [6]; furthermore, decent work is the ideal goal of working people and is an integral part of the International Labor Organization’s initiative aimed at integrating a social justice perspective into the working life of people around the world [7]. Based on these theoretical and practical calls, the psychology of working theory (PWT) [8] is proposed; it explains an individual’s career advancement by discussing contextual and socio-psychological factors simultaneously. To date, PWT is utilized to understand career behaviors of various groups, including working adults [9], marginalized grown-ups [10], and college students [11]. Few studies so far have examined constructs from the PWT with nursing college students, tested its application to a Chinese college sample, and linked the future decent work perceptions with students’ actual career exploration behaviors.

With a sample of nursing college students in China, we filled these gaps in the present study by connecting the antecedents of PWT with career exploration behavior, and observed the impact of economic resources on future decent work perceptions and career exploration behavior through two psychological factors (namely career adaptability and work volition) and explored whether a proactive personality moderated the impact of economic constraints on decent work perceptions, work volition, and career adaptability. Previous studies illustrated that a large number of nursing college students come from a middle to lower socioeconomic family background [12,13], and lots of them are prepared to work after graduation [14], but at the same time, they also encounter many difficulties and even discriminations in the labor market compared to their university counterparts [15,16]. Consequently, their employment result is not ideal, although they have strong employment willingness and hold high future expectations [14]. This study advances the PWT theory in the Chinese context in general, with nursing college students in particular; the perceived decent work is the first empirically identified as the antecedent of career exploration behavior.

## 2. Literature Review

### 2.1. Psychology of Working Theory

Psychology of working theory (PWT) is a newly proposed framework that intends to understand the working experiences of people in general, and those who suffer from economic constraints and marginalization in particular. Decent work plays a central role in the model, and it has five components: safe working conditions, access to health care, sufficient compensation, free time and rest during the work week, and values match between working place and family [8]. Previous studies have tested the model in various countries, such as Brazil [17], South Korea [18], Switzerland [19], UK [20], Italy [21], Turkey [22], Portugal [23], and China [9], and in various sorts of respondents, such as low-income employees [24], people who suffer from Chiari malformation [25], racially and ethnically diverse employees [10], and emerging adults [11].

The model places emphasis on the impact of contextual and psychological variables on individuals’ decent work acquisition. Economic constraints and marginalization are two important contextual factors that could predict the decent work. Economic constraints is defined as the few economic resources such as household income and family wealth that make it difficult for people to get decent work [8]. In a recently published article, Duffy et al. further explained this concept and proposed a scale that is more suitable for what they intend to measure [26]. The economic constraints in the PWT mainly refer to the economic constraints across an individual’s entire life; instead of that at the present time (e.g., current income and financial difficulty for the time being) [27]. Marginalization refers to derogating a person (or a group of people) to a less powerful status in society [8] and mainly focuses on discriminating experiences within a particular domain, due to certain social status, such as gender and ethnic identity [26].

In addition to the direct influences from economic constraints and marginalization to decent work, PWT also hypothesizes that these direct effects are mediated by two psychological factors. One is work volition, which refers to views on career choices despite the obstacles or restrictions [28]; the other is career adaptability, which is defined as the psychological resources that people could use to handle and complete tasks which are related to the career plan’s formulation and implementation [29,30]. Studies found that most Chinese nursing college students are from a middle-to-lower class family background [12]. Their parental occupations are primarily as workers, farmers, and small businessmen, and the majority have a are primary, junior, and senior high school education level [12]. Similar situations can be found in other countries [13].

PWT framework also has some moderator variables, which could be used to design appropriate interventions to lessen the shock of economic constraints and marginalization on one’s career adaptability, work volition, and the capacity to acquire decent work [8]. The theory believes that individuals could play their agency in daily life, negotiate contexts, and step over contextual barriers, such as economic constraints [8]. Therefore, based on personality, developmental, and vocational psychology literature, three moderators were proposed: proactive personality, critical consciousness, and social support [8]. Proactive personality refers to the personal initiative that tends to actively influence the environment [31], and challenges the status quo instead of passively adapting to it [32]. Those who show high levels of proactive personality tend to have positive career coping styles [33], work related motivation, and job performance [34].

Critical consciousness is made up of critical reflection, political efficacy and critical action. Critical reflection refers to a detailed examination of social and structural elements that lead to societal inequalities; political efficacy describes the perceived ability to initiate social and political change; and critical action is defined as the actions that individuals or groups take to reduce or eliminate perceived inequalities [35]. Critical consciousness empowers marginalized people to defeat structural constraints and achieve positive outcomes [36], and many studies show that it has a close relation with factors in the career development domain, such as career commitment, vocational identity [37], and vocational expectations [38]. Longitudinal studies illustrate that critical consciousness could enhance marginalized youth career-related expectations and thus help them to get higher status jobs when they grow up [39,40].

Social support portrays the extent to which individuals feel the support from family members, friends, significant others, and the wider community when dealing with the pressures and adversities related to marginalization and economic difficulties [41]. Existing vocational theories, such as the social cognitive career theory [42] employs social support as the antecedent of career success. It is conducive to one’s self-efficacy [43] and outcome anticipations [44]. Therefore, perceived career barriers would decrease [45], and this is particularly important for marginalized groups, such as first-generation college students [46]. This study employed the predictor session of PWT and proactive personality as the moderater and tried to link it to career exploration behavior of nursing college students.

### 2.2. Career Exploration

Career exploration is a vital dimension in many career-related theories [47,48], and it helps individuals to establish a coherent career plan, pursue a meaningful career, manage rapid career changes, and address various changes in their working lives [3,49,50]. It is defined as purposeful behavior and cognition which provide information on occupations, jobs, and organizations that were not previously in the field of stimulation [48]. Stumpf et al. [48] further divided this factor into two dimensions: self-exploration and environmental exploration. The former refers to individuals increasing knowledge of themselves by exploring their inner attributes; the latter describes the individuals’ collecting behaviors in terms of work, organizations, and occupations [48].

From a traditional perspective, career exploration is regarded as an important stage in one’s career development process, and it is often seen among teenagers and young people [51,52]. Scholars argue that it often occurs in the early stages of one’s life which will influence future career choices [52]. Yet this linear view of the career process was soon challenged [53], and career exploration began to be viewed as a “recycling” process. One’s career development is nonlinear, dynamic, and flexible, for everyone will face a series of complicated career experiences in all stages of life. People need to re-explore and reconstruct their career roles in all life stages, and this is particularly the case for those who are now experiencing life transitions [54]. Thus, career exploration is now seen as a lifelong, ongoing project [55].

### 2.3. Empirical Research and Hypotheses Development

#### 2.3.1. The Antecedents of Decent Work

According the PWT, those who suffer from economic problems have great difficulties in making career-related choices. The proposed theoretical relationship has been empirically supported thus far [25,26]. Therefore, the study put forward the first hypothesis:

**Hypothesis** **1** **(H1).** *Economic constraints negatively influence work volition.*

Similarly, those who have economic difficulties also tend to have fewer career-related psychological resources that could be utilized to address career-related exploration and implementation. To date, this theoretical assumption has been partially confirmed [25], with a few exceptions [26]. Therefore, the study proposed the second hypothesis:

**Hypothesis** **2** **(H2).** *Economic constraints negatively influence career adaptability.*

The majority of studies so far have supported the positive relationship between work volition and career adaptability [26,56]. In other words, those who are able to make career-related decisions are much more likely to have relevant psychological resources to help them solve problems. Thus, the study presented the third hypothesis:

**Hypothesis** **3** **(H3).** *Work volition positively influences career adaptability.*

Based on the theory, people who have economic difficulties often have a lower level of decent work perceptions. No consensus has been reached on this theoretical assumption, with some supporting empirically [25] and a few exceptions [11,26]. Although according to the theory, the study poses the negative relationship between the two as the fourth hypothesis:

**Hypothesis** **4** **(H4).** *Economic constraints negatively influence future decent work perceptions.*

Those who have the capacity to make their own career-related decisions tend to have positive decent work opinions. The majority of studies support this assumption empirically [9,10,56]. Therefore, the study advanced the fifth hypothesis:

**Hypothesis** **5** **(H5).** *Work volition positively influences future decent work perceptions.*

In a similar vein, people who have lots of psychological resources tend to develop the positive future decent work perceptions, which have been generally empirically supported [24,25], with a few exceptions [56]. Thus, the study proposed the sixth hypothesis:

**Hypothesis** **6** **(H6).** *Career adaptability positively influences future decent work perceptions.*

#### 2.3.2. Linking Decent Work Perceptions with Career Exploration

Empirical studies have identified the impact of personality traits on individuals’ career exploration behavior [57,58,59]. For example, scholars found that openness to experience, agreeableness, and responsibility have a positive relationship with career exploration [57]. Similarly, they found that those who cherish a desire with anticipation, namely hope, tend to have a higher level of career exploration behavior [58]. Moreover, individuals who display a better career self-evaluation tend to illustrate a higher level of career exploration behavior [59].

In addition to the personal beliefs mentioned above, perceptions in general and psychological ones in particular, tend to have an impact on one’s career exploration [60,61]. Studies show that college students’ views on their academic fitness and the consistency between their own career expectations and those of their parents lead them to participate more in career exploration activities [60]. In addition, for established adults who already have jobs, if they are dissatisfied with their current job, they may participate in career self- and environmental-exploration related to external job opportunities [61]. Based on these discussions, the study put forth the seventh hypothesis:

**Hypothesis** **7** **(H7).** *Future decent work perceptions positively influence career exploration behaviors.*

**Figure 1:**
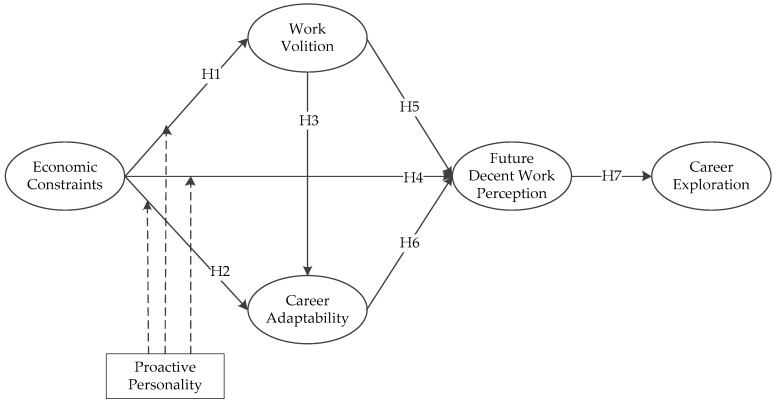
The hypothesized model.

Based on the reviewed literature, the theoretical framework in the study is proposed as Figure 1.

## 3. Research Method

### 3.1. Measurement Instruments

#### 3.1.1. Economic Constraints

Economic constraints were measured by the newly developed scale [26], which better assesses the economic constraint experience of one’s life. A sample item of the scale is: “Throughout most of my life, I have had fewer economic resources than most people”. Respondents were asked to rate on a seven-point Likert scale (1 = strongly disagree; 7 = strongly agree). The original reliability coefficient for the scale was 0.95 and our research was 0.92.

#### 3.1.2. Work Volition

Work volition was evaluated by the student version of Work Volition Scale [28]. Students were asked to evaluate the items from 1 = strongly disagree to 7 = strongly agree. Sample items include “I will be able to choose jobs if I want to”, and “I will learn how to find my own way in the world of work”. The original reliability of the scale was 0.86 and our research was 0.85.

#### 3.1.3. Career Adaptability

The Chinese version of the Career Adaptability Scale was used to measure students’ career adaptability [29]. It includes four factors, concern, control, curiosity, and confidence, and students were required to evaluate how strongly they have developed those abilities from 1 = not strong to 5 = strongest. Sample items include: “thinking about what my future will be like” (concern), “making decisions by myself” (control), “observing different ways of doing things” (curiosity), and “taking care to do things well” (confidence). Its validity and reliability have been tested well in previous studies [62,63]. The original and current reliability of the scale were 0.94 and 0.87, respectively.

#### 3.1.4. Future Decent Work Perceptions

The 15-item Decent Work Scale was utilized to assess decent work [64] and we followed previous studies and modified items to better understand nursing students’ perceptions [11]. Respondents were asked to evaluate the items (from 1 = strongly disagree to 7 = strongly agree) based on the following instructions, “Please consider the work/job that you will have in the future, and based on this image, rate the following items.” It contained five dimensions, and sample items were “at my future work, I will feel safe from emotional or verbal abuse of any kind” (safe working conditions), “my future employer will provide acceptable options for health-care” (access to healthcare), “I will be rewarded adequate for my future work” (sufficient compensation), “I will have no time to rest during the work week” (free time and rest), and “the values of my organization will match the values within my community” (complementary values). The internal consistency coefficient of the scale was 0.91 and our current one was 0.87.

#### 3.1.5. Career Exploration

The Chinese version of the Career Exploration Scale was employed to evaluate students’ career exploration [65]. Students were asked to assess the items (from 1 = never to 5 = always) based on the following instructions: “The following items are evaluations of your professional behavior in the past year. Please rate how often you considered these aspects in the past year”. Sample items include “I reflected on my past integrates with my future career” and “obtained information on the labor market and general job opportunities in my career area”. Cronbach’s alpha for the original and present study was 0.85 and 0.88, respectively.

### 3.2. Data Collection

A cross-sectional research design was employed, and the data were collected from one health vocational college situated in Northwest China. An electronic form of the questionnaire was used to collect the data. All the questions had to be answered before the questionnaire could be submitted, this ensured no missing data. We excluded those who reported careless answers (e.g., ages are 12121, 333, 12, 76). The final sample consisted of 854 individuals with a mean age of 20.21 years (ranged from 17 to 25 years old, SD = 1.55). The respondents’ demographic details are shown in Table 1.

## 4. Data Analysis

Data analysis followed the two-step method of structural equation modeling to evaluate the measurement and structural model [66]. The measurement model uses confirmatory factor analysis (CFA) to assess reliability and validity. The structural model tests the path effects and proposed significance. Moreover, analyses of the mediation and moderation effects were also performed.

### 4.1. Measurement Model Testing

Measurement model testing was performed in the following four steps. First, we evaluated the data’s reliability. According to the standards suggested in the literature, if the critical value (Cronbach’s alpha and composite reliability) is over 0.7 [67], the data indicated a fairly good reliability. We then performed the convergent validity tests. If the factor loading is over 0.5 or 0.6, composite reliability is over 0.7, and the average of variance extracted (AVE) is larger than 0.5 [67,68,69], the data showed good convergent validity. Table 2 illustrates the reliability and convergent validity of our data, and all critical values are met.

Second, we performed discriminant validity tests. According to Fornell and Larcker [69], if the square root of the AVE is larger than the correlations of each construct, the criterion of discriminant validity has been fulfilled. Table 3 shows that the smallest square root of the AVE in our data is 0.77, and it is larger than the largest correlation (0.62), indicating our data has passed the discriminant validity evaluation.

Third, the goodness-of-fit of the measurement model was tested. The second and third columns of Table 4 demonstrate the acceptable levels of criteria suggested in the literature and our own results. It showed that our results were above the critical values of the suggested literature, indicating the measurement model had a good fit with the empirical data.

### 4.2. Structural Model Testing

**Figure 2:**
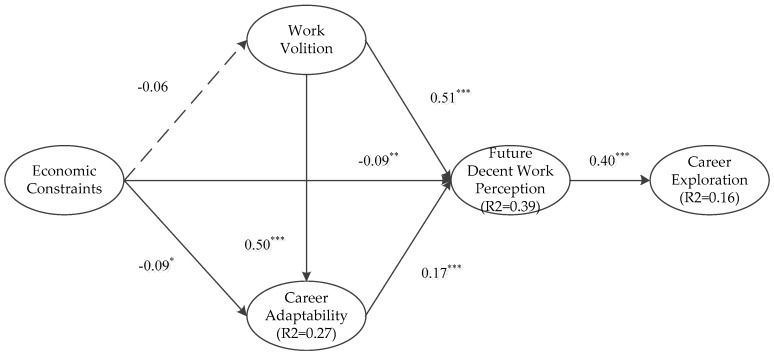
Standardized solution of structural modeling analysis (* *p*-value < 0.05; ** *p*-value < 0.01; *** *p*-value < 0.001).

The hypothesized relationships in the theoretical framework were tested by performing a structural model evaluation. The second and forth columns of Table 4 show the literature suggested acceptable model indicators and our own structural model index. It indicates that all these indicators of the structural model reached the satisfactory level. In addition, Table 5 displays the hypotheses testing results by illustrating standardized path coefficients and *t*-value. All hypothesized relationships are well confirmed, except the proposed negative relationship between economic constraints and work volition which was not supported in the data. Figure 2 shows the hypotheses testing results.

### 4.3. Testing of Mediating Effects

In order to fully understand the significance of the indirect effect, we performed the 95% confidence interval by bootstrapping. If zero is not in the interval, the mediation effect can be confidently confirmed [70,71]. Mediation effects were assessed using eight equations (Table 6). Since Hypothesis 1 (EC-WV: beta = −0.06, *t*-value = −1.67, *p* > 0.05) is not statistically significant, the indirect paths that contain H1 are not statistically significant either. In other words, H6a (EC-WV-FDP), H6e (EC-WV-FDP-CE), H6f (EC-WV-CA-FDP), and H6h (EC-WV-CA-FDP-CE) are not statistically significant. The rest of the indirect paths are statistically significant.

### 4.4. Testing of Moderating Effects

In order to assess the moderating effect of proactive personality, we performed the two-step analysis. First, we standardized all the observed variables of two factors: economic constraints and proactive personality. Second, we established the interaction factors by multiplying these standardized observed variables. Economic constraints have four items, and proactive personality has three items, and therefore, we got twelve items. Among the three theoretically supported moderation effects, namely, economic constraints **→** work volition (*t*-value = −0.59, *p*-value = 0.56), economic constraints **→** career adaptability, and economic constraints **→** future decent work perceptions (*t*-value = −0.03, *p*-value = 0.36), proactive personality only moderated the effects from economic constraints to the career adaptability, and its effect was 0.05 (*t*-value = 1.97, *p*-value = 0.05). The other two are not statistically significant.

## 5. Discussion

Based on the PWT, this study proposed an extended model, linking the predictor session of decent work with the career exploration behavior of nursing college students. Through empirical testing, the majority of the hypotheses of the model were supported. Firstly, H1 showed that economic constraints had a negative relationship with work volition, which means that students who suffered economic difficulties usually had a lower level of ability to make career-related decisions. The result, however, did not reach statistically significant, and this is not in line with the current findings [25,26]. The primary reason for this may be that a newly-proposed economic constraints scale was employed, and it focused on lifelong economic hardships instead of current financial difficulties that many related empirical studies utilize [25].

Secondly, H2 indicated that economic constraints have a negative relationship with career adaptability, which suggested that people who had few economic resources tend to have few psychological resources that could help them to overcome career transitions and traumas. This is consistent with other empirical findings [25], which confirmed the negative relationship between the two. Thirdly, H3 revealed that people who were able to make career-related decisions by themselves also had psychological resources that could assist them with career difficulties, which is consistent with the findings of Douglass et al. [56] and Kozan et al. [24]. Fourthly, H4 demonstrated that one’s economic conditions negatively impact their decent work perceptions, which empirically validates the findings of Tokar and Kaut [25].

H5 showed that individuals who were able to make career-related decisions by themselves held positive future decent work perceptions. This is in line with the majority of studies [9,11,56]. H6 indicated that career adaptability had a positive impact on future decent work perceptions, which illustrated that with a higher level of career adaptability, nursing college students had better views on their future careers. This is also in line with other studies [24,25]. Finally, H7 illustrated that nursing college students who are confident in their abilities to acquire decent work in the future tend to display better career exploration behavior. In other words, future decent work perceptions of nursing college students had a positive impact on their career exploration behavior. This is one of the major findings of this study, and it contributed another perception antecedent, decent work perception, to the career exploration literature.

## 6. Contributions, Implications, and Future Research Directions

The theoretical contributions of the current study can be summarized as follows. First, the PWT was first empirically tested among nursing college students. Specifically, we examined the antecedents of decent work of the PWT and linked them to career exploration behavior. Previous studies of the PWT model were performed mainly in Western cultures [19,20] and few of them focused on the Chinese context [9]. Our work enriches the applicable case of the PWT model. Second, previous studies have identified several perceptions variables that impact career exploration behavior [60,61]; our work contributed another vital antecedent of career exploration behavior, namely future decent work perceptions. The positive relationship between the two factors sheds some light on the future managerial practices which will be discussed in the coming session. In addition, the mediation effect of “EC→CA→FDP→CE” is substantiated for the first time, and therefore career counselors and educators could design appropriate interventions to enhance nursing college students’ career exploration behaviors and promote their foreseeable future.

The managerial implications for the practitioners, including professional educators and administrators, are as follows. First, the findings take specific meanings for nursing college students, the majority of whom are from a middle to lower socioeconomic family background [12,13], and many of them are prepared to enter the labor market soon after graduation [14]. In this case, it is of great significance for these students to develop positive future career opinions before they display the career-related exploration behaviors. Thus, career counselors who work with this group could benefit from the findings and design appropriate programs to enhance career exploration behavior. Second, economic constraints had a negative relationship with career adaptability and future decent work perceptions. As such, universities and career counselors could provide some opportunities to nursing students in general, in particular those suffering from economic problems, and help them to alleviate their economic conditions. These chances include, but are not limited to, paid internships on campus. Finally, the mediation effects of career adaptability might inform appropriate interventions. Career adaptability is seen as a moldable psychological construct, and because of this, in order to enhance career adaptability, counselors could focus on the four aspects of adaptability [29] and evaluate the nursing college students’ strengths and weakness in these aspects. Proper interventions based on the assessment could be designed and implemented accordingly.

Moreover, there are some limitations in this study in which future work could be enhanced from the following perspectives. First, the study employed a cross-sectional design, and thus the casual relations between the variables are difficult to confirm. Future studies could utilize a stricter research design, such as time-lagged panel studies, to establish a causal effect [72]. Second, the findings of this work must be carefully explained when they give general applicability to nursing students in other settings. The students in nursing professional colleges are all career-orientated, which is quite different from those who attend the nursing faculty in universities, many of whom are probably academic-oriented [14,73]. Future studies could collect data from more diverse backgrounds to improve further the external validity of the findings. Finally, the mechanism between decent work and career exploration behaviors is not clearly identified. Future studies should introduce some variables, such as employability [74], to further explore the relationship between the two.

## 7. Conclusions

In a word, the current study lent support for the application of the antecedent session of the PWT model among Chinese nursing college students when making appropriate changes to better suit the developmental phase of these students. Admittedly, this study has some limitations, but it casts light on the theoretical implications of applying the PWT on nursing college students in the Chinese context, and practical implications for professional career counselors and educators who work with this group and would like to improve their career exploration behaviors.

## Figures and Tables

**Table 1:** Sample demographics.

Characteristics	Frequency	Percent (%)
University Grade		
Freshman	146	17.1%
Sophomore	323	37.8%
Junior	158	18.5%
Senior	227	26.6%
Place of Origin		
Rural	609	71.3%
Urban	245	28.7%
Parental Educational Level		
Primary certificate or below	149	17.4%
Junior high school	320	37.5%
Senior high school	247	28.9%
Undergraduate degree or above	138	16.2%
Gender		
Male	177	20.7%
Female	677	79.3%

**Table 2:** Standardized loadings and reliabilities.

Construct	Indicators	Factor Loading	Composite Reliability	Cronbach’s α	AVE
EC	EC1	0.88	0.93	0.92	0.76
	EC2	0.92	--	--	--
	EC3	0.77	--	--	--
	EC4	0.90	--	--	--
WV	WV1	0.76	0.85	0.85	0.59
	WV2	0.74	--	--	--
	WV3	0.73	--	--	--
	WV4	0.84	--	--	--
CA	CA1	0.68	0.87	0.87	0.63
	CA2	0.84	--	--	--
	CA3	0.77	--	--	--
	CA4	0.87	--	--	--
FDP	FDP1	0.56	0.88	0.87	0.59
	FDP2	0.76	--	--	--
	FDP3	0.89	--	--	--
	FDP4	0.81	--	--	--
	FDP5	0.78	--	--	--
CE	CE1	0.76	0.88	0.88	0.66
	CE2	0.73	--	--	--
	CE3	0.91	--	--	--
	CE4	0.83	--	--	--

Note: AVE: average of variance extracted; EC: economic constraints; WV: work volition; CA: career adaptability; FDP: future decent work perceptions; CE: career exploration.

**Table 3:** Correlation coefficient matrix and discriminant validity.

	Mean	SD	AVE	CE	FDP	CA	WV	EC
CE	3.50	0.87	0.66	**0.81**				
FDP	4.79	1.17	0.59	0.37	**0.77**			
CA	3.87	0.78	0.63	0.62	0.41	**0.79**		
WV	4.59	1.25	0.59	0.45	0.59	0.51	**0.77**	
EC	4.31	1.65	0.76	−0.16	−0.14	−0.12	−0.06	**0.87**

Note: EC: economic constraints; WV: work volition; CA: career adaptability; FDP: future decent work perceptions; CE: career exploration. Off diagonals are Pearson’s correlation of constructs. Square root of AVE in bold on diagonals.

**Table 4:** Goodness-of-fit indices for the measurement scales.

Fit Index	Recommended Value	Measurement Model	Structural Model
Chi-squared/df	≤5	2.72	4.02
GFI	≥0.90	0.95	0.92
CFI	≥0.92	0.97	0.95
NFI	≥0.90	0.96	0.93
IFI	≥0.90	0.97	0.95
TLI	≥0.90	0.97	0.94
RFI	≥0.90	0.95	0.92
PGFI	≥0.50	0.73	0.73
PCFI	≥0.50	0.83	0.82
PNFI	≥0.50	0.82	0.81
RMSEA	≤0.08	0.04	0.06

Note: GFI = goodness-of-fit index, CFI=comparative fit index, NFI = normed fit index, IFI = incremental fit index, TLI = Tucker Lewis index, RFI=relative fit index, PGFI = parsimony goodness-of-fit index, PCFI = parsimony comparative-of-fit index, PNFI = parsimony normed fit index, RMSEA = the root mean square error of approximation.

**Table 5:** Summary of hypotheses testing results.

Hypotheses		Standardized Path Coefficients	*t*-Value	Supported
H1	EC → WV	−0.06	−1.67	No
H2	EC → CA	−0.09 *	−2.59	Yes
H3	WV → CA	0.50 ***	12.72	Yes
H4	EC → FDP	−0.09 **	−2.88	Yes
H5	WV → FDP	0.51 ***	10.18	Yes
H6	CA → FDP	0.17 ***	4.11	Yes
H7	FDP → CE	0.40 ***	9.26	Yes

Note: EC: economic constraints; WV: work volition; CA: career adaptability; FDP: future decent work perceptions; CE: career exploration. * *p*-value < 0.05; ** *p*-value < 0.01; *** *p*-value < 0.001.

**Table 6:** Mediation tests.

Parameter		Estimate	Lower	Upper	*p-*Value
H6a	EC-WV-FDP	−0.018	−0.042	0.004	0.113
H6b	EC-CA-FDP	−0.008	−0.018	−0.002	0.003
H6c	EC-FDP-CE	−0.018	−0.035	−0.004	0.007
H6d	WV-CA-FDP	0.064	0.028	0.100	0.002
H6e	EC-WV-FDP-CE	−0.006	−0.015	0.001	0.110
H6f	EC-WV-CA-FDP	−0.003	−0.008	0.001	0.089
H6g	EC-CA-FDP-CE	−0.003	−0.007	−0.001	0.004
H6h	EC-WV-CA-FDP-CE	−0.001	−0.003	0.000	0.079

Note: EC: economic constraints; WV: work volition; CA: career adaptability; FDP: future decent work perceptions; CE: career exploration.
